# Gut microbiota modulates differential lipid metabolism outcomes associated with FTO gene polymorphisms in response to personalized nutrition intervention

**DOI:** 10.3389/fnut.2022.985723

**Published:** 2022-09-15

**Authors:** Jianheng Zheng, Feijie Wang, Hongwei Guo, Junrui Cheng, Jun Du, Juntao Kan

**Affiliations:** ^1^Nutrilite Health Institute, Shanghai, China; ^2^School of Public Health, Fudan University, Shanghai, China; ^3^Department of Molecular and Structural Biochemistry, North Carolina State University, Raleigh, NC, United States

**Keywords:** personalized nutrition, lipid metabolism, RCT, *FTO*, gut microbiota

## Abstract

**Background:**

Interindividual differences in response to personalized nutrition (PN) intervention were affected by multiple factors, including genetic backgrounds and gut microbiota. The fat mass and obesity associated (*FTO*) gene is an important factor related to hyperlipidemia and occurrence of cardiovascular diseases. However, few studies have explored the differences in response to intervention among subjects with different genotypes of *FTO*, and the associations between gut microbiota and individual responses.

**Objective:**

To explore the differential lipid metabolism outcomes associated with *FTO* gene polymorphisms in response to PN intervention, the altered taxonomic features of gut microbiota caused by the intervention, and the associations between gut microbiota and lipid metabolism outcomes.

**Methods:**

A total of 400 overweight or obese adults were recruited in the study and randomly divided into the PN group and control group, of whom 318 completed the 12-week intervention. The single nucleotide polymorphism (SNP) of rs1121980 in *FTO* was genotyped. Gut microbiota and blood lipids were determined at baseline and week 12. Functional property of microbiota was predicted using Tax4Fun functional prediction analysis.

**Results:**

Subjects with the risk genotype of *FTO* had significantly higher weight and waist circumference (WC) at baseline. Generalized linear regression models showed that the reduction in weight, body mass index (BMI), WC, body fat percentage, total cholesterol (TCHO), and low-density lipoprotein (LDL) was greater in subjects with the risk genotype of *FTO* and in the PN group. Significant interaction effects between genotype and intervention on weight, BMI, WC, TCHO, and LDL were found after stratifying for specific genotype of *FTO*. All subjects showed significant increasement in α diversity of gut microbiota after intervention except for those with the non-risk genotype in the control group. Gut microbiota, including *Blautia* and *Firmicutes*, might be involved in lipid metabolism in response to interventions. The predicted functions of the microbiota in subjects with different genotypes were related to lipid metabolism-related pathways, including fatty acid biosynthesis and degradation.

**Conclusion:**

Subjects with the risk genotype of *FTO* had better response to nutrition intervention, and PN intervention showed better amelioration in anthropometric parameters and blood lipids than the control. Gut microbiota might be involved in modulating differential lipid metabolism responses to intervention in subjects with different genotypes.

**Trial registration:**

[Chictr.org.cn], identifier [ChiCTR1900026226].

## Introduction

Hyperlipidemia is a common risk factor of cardiovascular diseases (CVD), which are the predominant cause of mortality and healthcare expenditure worldwide (1). Global Burden of Disease Study showed that low-density lipoprotein (LDL) has contributed to the increasing disease burden since 1990 and has become the sixth risk factor for death in the Chinese population (2). Cardiovascular diseases result from the combined additive and synergistic effects of genetic and environmental risk, including diet and physical activity (PA) (3). Numerous strategies have been developed to reduce the risk of CVD, in which dietary intervention is of great importance (4).

With the rising prevalence of chronic diseases in recent years, personalized nutrition (PN) has received increasing attention, which incorporates one's personal and cultural preferences as well as genetic traits to create the most appropriate and effective wellness strategies (4, 5). Dietary intervention studies have found that responses varied dramatically among individuals in a group, while interindividual differences in response to diet are poorly understood (6). Previous studies suggested that genetic variation could modulate the response to diet, and multi-locus genotype patterns were relevant to lipid metabolism (7). Of note, the fat mass and obesity-associated (*FTO*) gene is one of the most known genetic factors predisposing humans to non-monogenic obesity, and the *FTO* SNP variant has the strongest known effect on increased BMI and body fat (8, 9), which could be the potential risk factor for disturbing lipid metabolism and increasing the risk of CVD (10).

A meta-analysis of 10 studies and 6,951 participants showed that individuals with risk allele of *FTO* could lose more weight through diet/lifestyle interventions than non-carriers (11). The underlying mechanisms are unclear, but it has been suggested that gut microbiota might participate in the differential response to dietary intervention (12). Previous studies have proved that enterotypes could affect the outcomes of diet intervention. For instance, *Prevotella* has been shown to be associated with improved glucose metabolism after barley kernel-based bread intervention (13). In addition, deletion of *FTO* led to lower body weight in mice, and behavioral alterations of *FTO* +/- mice were mediated by shift in gut microbiota (14). However, to our knowledge, no studies have explored the mechanism of interindividual differences in response to intervention in populations with different genotypes of *FTO* from the perspective of gut microbiota.

Consequently, this study aims to determine the difference in changes of lipid metabolism in population with different genotypes of *FTO* after PN intervention based on a randomized controlled trial (RCT) conducted in Chinese overweight adults. Further, the association between interindividual difference and gut microbiota was explored. We believe the current study helps to elucidate the mechanism of differential response to interventions and pave the road toward personalized clinical therapy.

## Methods

### Study design

This study was a multicenter RCT with 2-group parallel design conducted in Shanghai Aier Hospital and Songnan Hospital, China. Detailed information has been described previously (15). This trial was approved by the Institutional Review Board (IRB) of the Shanghai Nutrition Society and registered at chictr.org.cn (ChiCTR1900026226).

### Participants

Subjects aged 25 to 50 years with BMI ≥ 24 kg/m^2^ were recruited in this study after signing informed consent form. The major criteria for exclusion were having coronary heart disease, cerebrovascular disease, liver disease, kidney disease, inflammatory bowel disease, or hematologic disease; being pregnant or lactating; taking any drugs or dietary supplements in recent days, which could interfere with the study results; or participating in another clinical trial in the past 3 months.

A total of 400 participants were initially recruited in the study at baseline, and randomly divided into PN group and control group at a 1:1 ratio using the Random Sequence Generator (https://www.random.org/sequences/). Participants did not learn of their group assignment until they were determined eligible for enrollment. The flow of the participants through the trial has been described previously (15).

### Dietary and physical activities information collection

Dietary information of participants was obtained by semiquantitative food frequency questionnaire (FFQ) at baseline and the end of week 12. Meanwhile, 24-h dietary recall (24-h), which was used to calculate daily energy and nutrients intake, and sport frequency questionnaire (SFQ), which was used to obtain the PA information, were conducted every 2 weeks. The metabolic equivalents of task (METs)-min/week were obtained by multiplying the average energy expenditure by min/week for each PA intensity (16). In addition, participants were asked to wear a sport band every day to collect daily exercise data. Anthropometric measurements, including weight, body mass index (BMI), body fat percent, and waist circumference (WC), were conducted at baseline and week 12.

### Intervention

The intervention in both groups lasted for 12 weeks, which has been described in detail elsewhere (15). Briefly, for the control group, participants were given traditional health guidance on diet, PA, and nutritional supplements intake per *Dietary Guidelines for Chinese Residents and Chinese DRIs Handbook* by means of a brochure provided at baseline.

For the PN group, an integrated personalized solution including recommendations on diet, PA, and dietary supplements (Nutrilite, Guangzhou, China) was generated by registered dietitians based on decision trees (15), which was modified from the Food4Me study decision trees (17). First, a PN report was generated based on the data collected at baseline, including individual's anthropometric, nutrient, blood, and genetic (nutrition-related) profiles. The results of these data were categorized into three tiers: normal, moderately higher or lower, and severely higher or lower. Second, 3~5 targeted goals were identified by registered dietitians for each participant according to the report, along with specific recommendations. For instance, if a person's vitamin C was below the recommended value, he or she was advised to consume more vitamin C-rich foods, such as fruit. Additional nutritional supplement was suggested when the calculated intake of a certain nutrient was lower than 80% of the estimated average requirement (EAR) and a risk allele was observed. If a weight loss goal was set, energy-restricted diet was recommended, with an additional recommendation for physical activity. The PN report and advice were biweekly updated according to the 24-h and PA assessments during the study.

### Genotyping

After recruitment, buccal cells of eligible participants were collected using oral swab (DNA Genotek, Ottawa, ON, Canada), with DNA extracted and analyzed by WuXi NextCode (Shanghai, China) using an Asian SNP Screening Chip (Illumina, San Diego, California, USA). The single nucleotide polymorphism (SNP) of rs1121980 in *FTO* was genotyped. The rs1121980 was significantly associated with morbid obesity (18), non-alcoholic fatty liver disease (19), and gestational diabetes mellitus (20) in Chinese after adjusting for potential confounders. The risk allele of *FTO* was T, and the genotype was classified into risk genotype (TT and TC) and non-risk genotype (CC).

### Blood lipids measurements

Fasting peripheral blood was collected at baseline and week 12, and serum was immediately separated after centrifugation. Four kinds of blood lipids were analyzed by colorimetry in this study: the levels of total cholesterol (TCHO), triglycerides (TG), LDL, and high-density lipoprotein (HDL).

### Fecal collection and 16S RRNA sequencing

At baseline and week 12, fresh fecal samples were collected from all participants using stool specimen container. Fecal samples were frozen at −20°C immediately after collection, and then stored at−80°C until DNA extraction. Total DNA from fecal samples was extracted using QIAamp DNA Stool Mini Kit following manufacturer's protocol (QIAGEN, Hilden, Germany). Subsamples from the DNA extracts were used for amplifying the V3-V4 region of the microbial 16S rRNA gene by using primers 341F (CCTACGGGNGGCWGCAG) and 805R (GACTACHVGGGTATCTAATCC) (402,425 reads), followed by sequencing by Illumina HiSeq 3000. The sequencing raw data was optimized using Cutadapt (v1.9.1), Vsearch (1.9.6) and Qiime 1 (1.9.1) software. The trimmed reads were clustered into Operational Taxonomic Units (OTUs) at 97% identity level using Qiime 1 and Vsearch software. The taxonomy of each OTU representative sequence was analyzed by RDP Classifier 2.2 (http://rdp.cme.msu.edu/) with 70 % as confidence threshold using the Silva database (SSU128). OTU abundance information was normalized using a standard sequence number corresponding to the sample with the least sequences.

Gut microbial α diversity (Shannon index) in subjects with different genotypes at baseline and Week 12 was calculated using the Vegan package (version 2.6-2) in R (version 4.2.0, R Core Team). The significant taxonomic features were performed using linear discriminant analysis (LDA) effect size (LEfSe) in galaxy software (http://huttenhower.sph.harvard.edu/galaxy/) with an effect logarithmic LDA score threshold of 2.0 and *p*-value threshold of 0.05. Functional property of microbiota was predicted using the Tax4Fun2 package in R, and the significant pathways related to lipid metabolism were illustrated with STAMP software. The low-abundance filters were applied to discard taxonomic and functional features whose relative abundance did not reach 0.1 and 0.001%, respectively.

### Statistical analyses

The per-protocol (PP) population who completed the study and provided blood samples at the end of the study with available genotype information was considered the primary analysis populations for the evaluation. Continuous variables with a normal distribution were presented as mean ± standard deviations (SD), highly skewed data were presented as median and quartiles, and categorical variables were presented as frequencies and percentages. Non-normal continuous outcomes were transformed using the natural logarithm (Ln) to improve the normality in the following analysis. Differences of demographics and anthropometric parameters between the risk genotype of *FTO* group and non-risk genotype group were assessed using *t* test for continuous variables and chi-square test for categorical variables. Group difference of diet and PA at baseline was evaluated using one-way analysis of variance (ANOVA); the difference at week 12 was evaluated using analysis of covariance (ANCOVA) with adjustment for the baseline measures; within group change from baseline to week 12 was evaluated using paired *t*-test. Difference of baseline lipids in different specific genotype of *FTO* groups was evaluated using ANOVA, and Bonferroni adjustment was used for pairwise comparisons.

Associations between change of anthropometric parameters and blood lipids from baseline to week 12 with genotype with the intervention group were assessed by generalized linear regressions (GLM) with the non-risk genotype and control group set as reference. A thousand times of bootstrap were used to generate 95% confidence interval (CI) of coefficients. Model 1 only included genotype and intervention group as independent variables, and Model 2 further adjusted for age, BMI, intake of macronutrients, total MET, and anthropometric or lipid measurements at baseline. In addition, possible interactions between genotype and intervention were investigated by introducing the corresponding interaction terms in Model 3. After stratifying for specific genotypes, the change of blood lipids in different genotypes or in two intervention groups was assessed by Mann-Whitney U Test with Bonferroni adjustment for pairwise comparisons. Associations between the change of blood lipids with genotype and intervention were assessed by GLM as described above.

Statistical analyses were performed using R version 4.2.0 (R Core Team). All statistical tests were 2-sided and conducted at the 0.05 level of significance.

## Results

### Characteristics of study population

A total of 400 participants were screened for inclusion from the initially recruited 2,718 participants, of whom 318 (79.5%) completed the 12-week intervention (41.2% were males with the average age of 38.4 years, with no significant difference in the PN group and the control group).

For the 318 subjects who completed the trial, the average body weight, BMI, WC, and body fat percent at baseline were 73.8 kg, 27.2 kg/m^2^, 92.6 cm, and 27.5% in the PN group and 72.7 kg, 27.3 kg/m^2^, 92.4 cm, and 27.2% in control group, showing no significant difference. The average TCHO, TG, HDL, and LDL at baseline were 5.28, 1.51, 2.94, and 1.51 mmol/L in the PN group and 5.21, 1.51, 2.93, and 1.36 mmol/L in the control group, respectively, with no statistically significant difference.

The proportion of subjects with the risk genotype of *FTO* (34.0%) was significantly lower than the proportion of subjects with the non-risk genotype (66.0%). After stratifying for genotypes of *FTO*, the baseline demographics, anthropometric parameters, and blood lipids of study population are shown in Table 1. Subjects with the risk genotype had significantly higher weight (*p* = 0.021) and WC (*p* = 0.003) at baseline than those with the non-risk genotype in the whole population and in the PN group. Subjects with the risk genotype had significantly lower HDL concentrations than those with the non-risk genotype in the control group (*p* = 0.045).

**Table 1:** Baseline demographics, anthropometric parameters, and blood lipids in different genotype groups.

**Factor**	**PN group (*****N*** = **166)**				**Control group (*****N*** = **152)**			
	**Risk genotype**	**Non-risk genotype**		***p*** **-value^a^**	**Risk genotype**	**Non-risk genotype**		***p*** **-value^a^**
	**TT (*N* = 5)**	**TC (*N* = 49)**	**CC (*N* = 112)**		**TT (*N* = 4)**	**TC (*N* = 50)**	**CC (*N* = 98)**	
Age (year)	35.2 ± 9.42	38.8 ± 8.24	38.9 ± 7.15	0.735	35.5 ± 3.87	37.5 ± 7.06	38.5 ± 7.76	0.382
Gender [male, No. (%)]	4 (80.0)	24 (49.0)	43 (38.4)	0.101	3 (75.0)	23 (46.0)	34 (34.7)	0.104
Weight (kg)	84.9 ± 8.22	76.3 ± 13.4	72.2 ± 11.1	**0.021**	79.1 ± 7.20	74.3 ± 11.8	71.6 ± 9.96	0.087
BMI (kg/m^2^)	28.7 ± 4.76	27.5 ± 2.85	27.0 ± 2.74	0.229	27.0 ± 1.20	27.8 ± 4.34	27.0 ± 2.80	0.188
WC (cm)	101.4 ± 2.51	95.2 ± 9.01	91.1 ± 9.50	**0.003**	91.3 ± 6.22	94.3 ± 12.4	91.6 ± 8.46	0.181
Body fat (%)	26.2 ± 7.89	28.1 ± 5.24	27.3 ± 4.98	0.436	27.8 ± 7.20	27.0 ± 5.82	27.3 ± 5.07	0.792
TCHO (mmol/L)	5.73 (4.69, 6.61)	4.97 (4.45, 5.83)	5.33 (4.68, 5.92)	0.565	5.32 (5.17, 6.98)	5.15 (4.56, 5.65)	5.17 (4.59, 5.78)	0.726
TG (mmol/L)	1.17 (1.05, 1.69)	1.28 (0.98, 1.78)	1.27 (0.85, 1.82)	0.467	1.34 (0.93,3.20)	1.08 (0.86, 1.68)	1.13 (0.80, 1.69)	0.916
HDL (mmol/L)	1.39 (1.28, 1.68)	1.44 (1.28, 1.59)	1.50 (1.34, 1.72)	0.075	1.29 (1.27, 1.58)	1.39 (1.28, 1.65)	1.51 (1.30, 1.77)	**0.045**
LDL (mmol/L)	3.62 (2.68, 3.97)	2.73 (2.37, 3.36)	2.82 (2.50, 3.48)	0.844	3.91 (2.78, 4.73)	2.70 (2.40, 3.22)	2.86 (2.46, 3.30)	0.732

a Difference between risk genotype and non-risk genotype was evaluated using t test for age and anthropometric parameters, chi-square test for gender, and Mann-Whitney U test for blood lipids. PN, personalized nutrition; BMI, body mass index; WC, waist circumference; TCHO, total cholesterol; TG, triglycerides; LDL, low-density lipoprotein; HDL, high-density lipoprotein. *P*-values less than 0.05 are marked in bold.

### Dietary intake and physical activity

The intake of energy and three macronutrients assessed by 24-h and PA of the participants are shown in Supplementary Table 1. Apart from total MET, diet intake and PA at baseline were not significantly different between the risk genotype group and non-risk genotype group. Participants with the non-risk genotype showed significantly lower fat intake (PN: *p* = 0.007; control: *p* = 0.029) and higher total MET (PN: *p* = 0.008; control: *p* = 0.001) after intervention in both PN group and control group. Besides, participants in the PN group showed significantly higher total MET (non-risk genotype: *p* = 0.008; risk genotype: *p* = 0.024) and daily walks (non-risk genotype: *p* < 0.001; risk genotype: *p* = 0.001) after intervention, regardless of genotype.

### Change of anthropometric parameters and blood lipids

Associations between genotype and intervention with change of anthropometric parameters and lipids from baseline to week 12 are shown in Supplementary Table 2. For anthropometric parameters, subjects with the risk genotype of *FTO* showed greater reduction in weight, BMI, WC, and body fat percent than those with the non-risk genotype. Subjects in the PN group showed greater reduction in all anthropometric parameters than those in the control group. For blood lipids, subjects with the risk genotype of *FTO* showed greater reduction in TCHO and LDL after intervention than those with the non-risk genotype. The amelioration of TCHO, TG, and LDL of populations in the PN group was significantly better than those in the control group.

After stratifying for specific *FTO* genotype, subjects with TT genotype had greater weight, BMI, and LDL reduction, and HDL increasement than those with TC or CC genotype in the control group as shown in Table 2. Subjects with TC genotype showed greater reduction in WC and fat percent than those with CC genotype. Subjects with TC genotype showed significantly greater reduction in weight (*p* = 0.032), BMI (*p* = 0.010), fat percent (*p* = 0.006), and TCHO (*p* = 0.019) than those with CC genotype in the PN group. In addition, significant interaction effects between genotype and intervention on weight, BMI, WC, TCHO, and LDL was found, indicating that intervention posed different effects on subjects with different genotypes.

**Table 2:** Change of anthropometric parameters and blood lipids from baseline to week 12 in subjects with different genotypes in control group or PN group.

**Lipid**	**PN group (*****N*** = **166)**			**Control group (*****N*** = **152)**			***P*** **-value**		
	**Risk genotype**		**Non-risk genotype**	**Risk genotype**		**Non-risk genotype**	**Genotype**	**Intervention**	**Genotype ×**
	**TT (*****N*** **= 5)**	**TC (*****N*** **= 49)**	**CC (*****N*** **= 112)**	**TT (*****N*** **= 4)**	**TC (*****N*** **= 50)**	**CC (*****N*** **= 98)**			**Intervention**
**Anthropometric parameters**									
Δ_Weight_ (kg)	−2.10 (-2.70,−1.45)	−3.10 (-3.85,−1.95)	−2.45 ^∧^(-3.70,−0.13)	−3.20 (-5.48,−2.35)	−1.65^*^ (-2.40,−0.88)	−1.35 ^#^ (-2.78, 1.60)	**0.036**	**<0.001**	**0.018**
Δ_BMI_ (kg/m^2^)	−0.78 (-0.88,−0.43)	−1.19 (-1.48,−0.73)	−0.89 ^∧^(-1.42,−0.02)	−1.18 (-1.77,−0.92)	−0.76^*^ (-0.92,−0.25)	−0.46 ^#^ (-1.05, 0.58)	0.061	**<0.001**	**0.046**
Δ_WC_ (cm)	−3.00 (-4.00,−2.00)	−3.00 (-5.00,−2.00)	−3.00 (-4.00,−1.00)	−3.60 (-6.30,−2.25)	−2.00 (-4.00,−1.00)	−1.00 ^#, ∧^(-3.00, 1.00)	**0.036**	**<0.001**	**0.034**
Δ_fat percent_ (%)	−1.20 (-3.15, 0.35)	−1.30 (-2.40,−0.45)	−0.70^∧^ (-2.00,−0.10)	−0.65 (-1.63,−0.20)	−0.50 (-1.50,−0.30)	−0.20^∧^ (-0.93, 0.70)	0.254	**<0.001**	0.402
**Blood lipids**									
Δ_TCHO_ (mmol/L)	−0.31 (-0.51,−0.02)	−0.38 (-0.60,−0.20)	−0.22^∧^ (-0.51,−0.01)	−0.29 (-2.39,−0.14)	−0.22 (-0.48, 0.06)	−0.06 (-0.33, 0.21)	0.061	**<0.001**	**0.010**
Δ_TG_ (mmol/L)	−0.13 (-0.65,−0.09)	−0.29 (-0.48,−0.10)	−0.23 (-0.41,−0.07)	−0.08 (-1.11, 0.21)	−0.10 (-0.35, 0.03)	−0.09 (-0.30, 0.09)	0.487	0.058	0.265
Δ_HDL_ (mmol/L)	0.14 (0.06, 0.31)	0.13 (0.06, 0.19)	0.12 (0.03, 0.19)	0.41 (0.25, 0.53)	0.11^**^ (-0.07, 0.23)	0.04 ^##^ (-0.07, 0.21)	**0.014**	0.057	0.052
Δ_LDL_ (mmol/L)	−0.53 (-0.68,−0.25)	−0.40 (-0.52,−0.33)	−0.41 (-0.60,−0.12)	−0.89 (-0.94, 0.64)	−0.42^**^ (-0.55,−0.01)	−0.24 ^##^ (-0.58, 0.15)	**0.022**	**0.024**	**0.032**

Data were presented as median and IQR. Difference between different genotypes was assessed by Mann-Whitney U Test with Bonferroni adjustment for pairwise comparisons: significance level 0.05/3 ≈ 0.017.

* : difference between TT and TC, ^*^p < 0.017,

** p < 0.003;

# : difference between TT and CC, ^#^ p < 0.017,

## p < 0.003;

∧ : difference between TC and CC, p < 0.017. The effects of genotype, intervention, and genotype × intervention interaction were evaluated using generalized linear models, adjusted for age, BMI, intake of macronutrients, total MET, and measurements at baseline.

### Association between gut microbiota with genotype and intervention effect

The taxonomic features between subjects with the risk genotype of *FTO* and with the non-risk genotype at baseline is shown in Supplementary Figure 1. The gut microbiota species with the greatest difference between the two groups were *Barnesiellaceae, Desulfobacterota* et al. in the subjects with the non-risk genotype and *NK4A214_group* et al. in those with the risk genotype, respectively.

**Figure 1:**
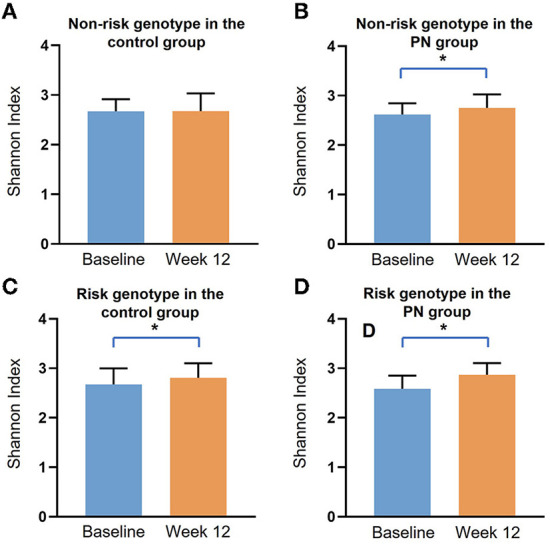
The α diversity of gut microbiota in subjects with different genotypes of *FTO* at baseline and week 12 in the control group or PN group. Data are presented as median and IQR, *: *p* < 0.05 by Mann-Whitney U test. **(A)** Non-risk genotype in the control group; **(B)** Non-risk genotype in the PN group; **(C)** Risk genotype in the control group; **(D)** Risk genotype in the PN group.

**Figure 2:**
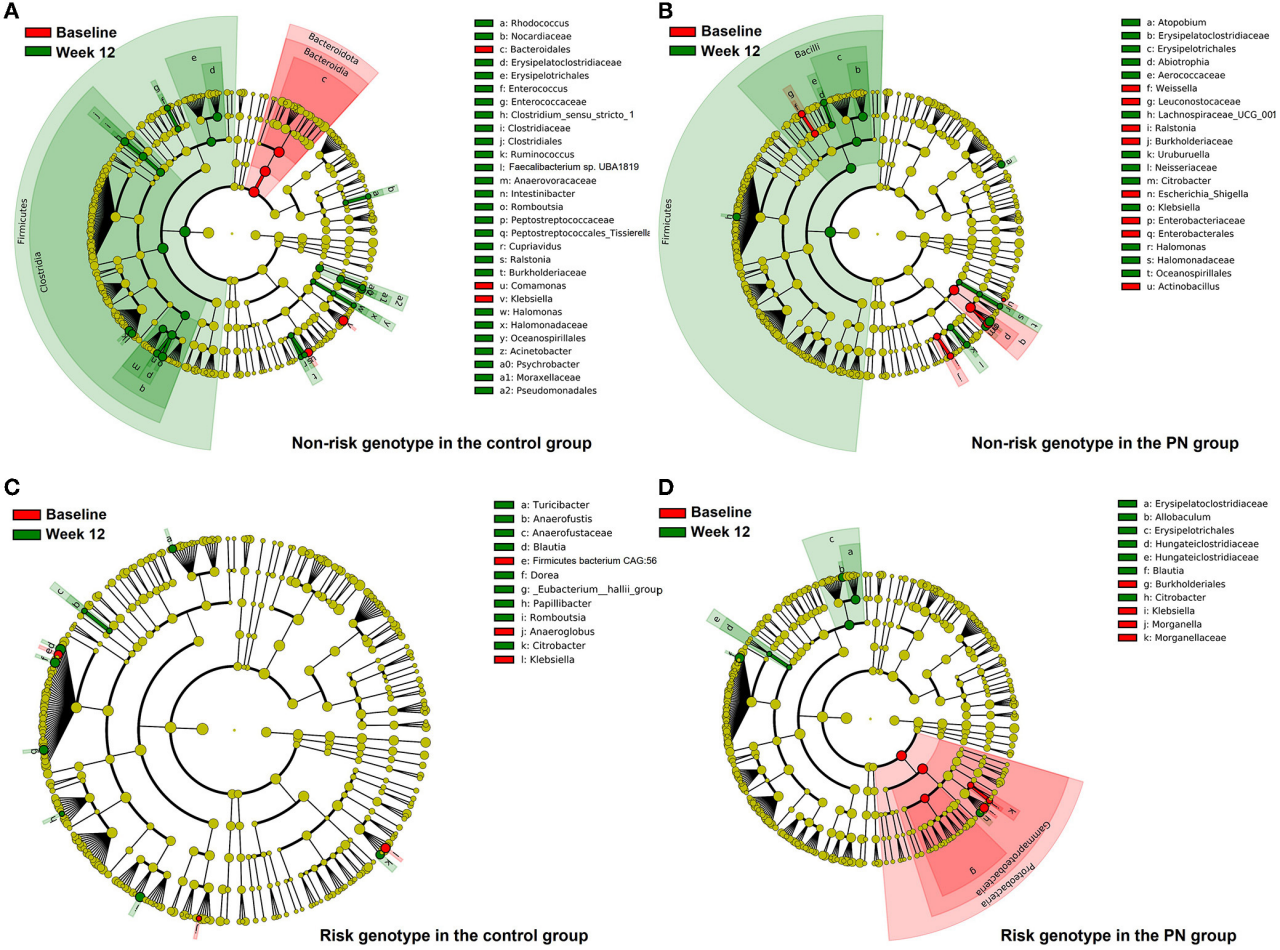
Comparison of gut microbiota composition before and after intervention in subjects with different genotypes of *FTO*. Taxonomic representation of statistically and biologically consistent differences between baseline (red) and Week 12 (green) shown in cladogram by LefSe analysis. Differences are represented by the color of the most abundant class. Dot size is proportional to the abundance of the taxon. **(A)** Non-risk genotype in the control group; **(B)** Non-risk genotype in the PN group; **(C)** Risk genotype in the control group; **(D)** Risk genotype in the PN group.

First, we explored the Shannon's diversity index of gut microbiota (Figure 1). It was found that all subjects had significant increasement of α diversity at week 12 except for those with the non-risk genotype in the control group. Next, we explored the specific microbiota with different expressions as shown in Figure 2. The cladogram showed that for the non-risk genotype subjects, 29 and 21 differentially abundant taxa were found in the control group and the PN group after intervention. For the risk genotype subjects, 12 and 11 differentially abundant taxa were found in the control group and the PN group after intervention. The significant taxonomic features of subjects with the non-risk genotype of *FTO* in control group or PN group between baseline and week 12 were shown in Supplementary Figures 2, 3. Both the control and PN intervention could increase *Firmicutes* in subjects with the non-risk genotype and *Blautia* in subjects with the risk genotype, respectively.

**Figure 3:**
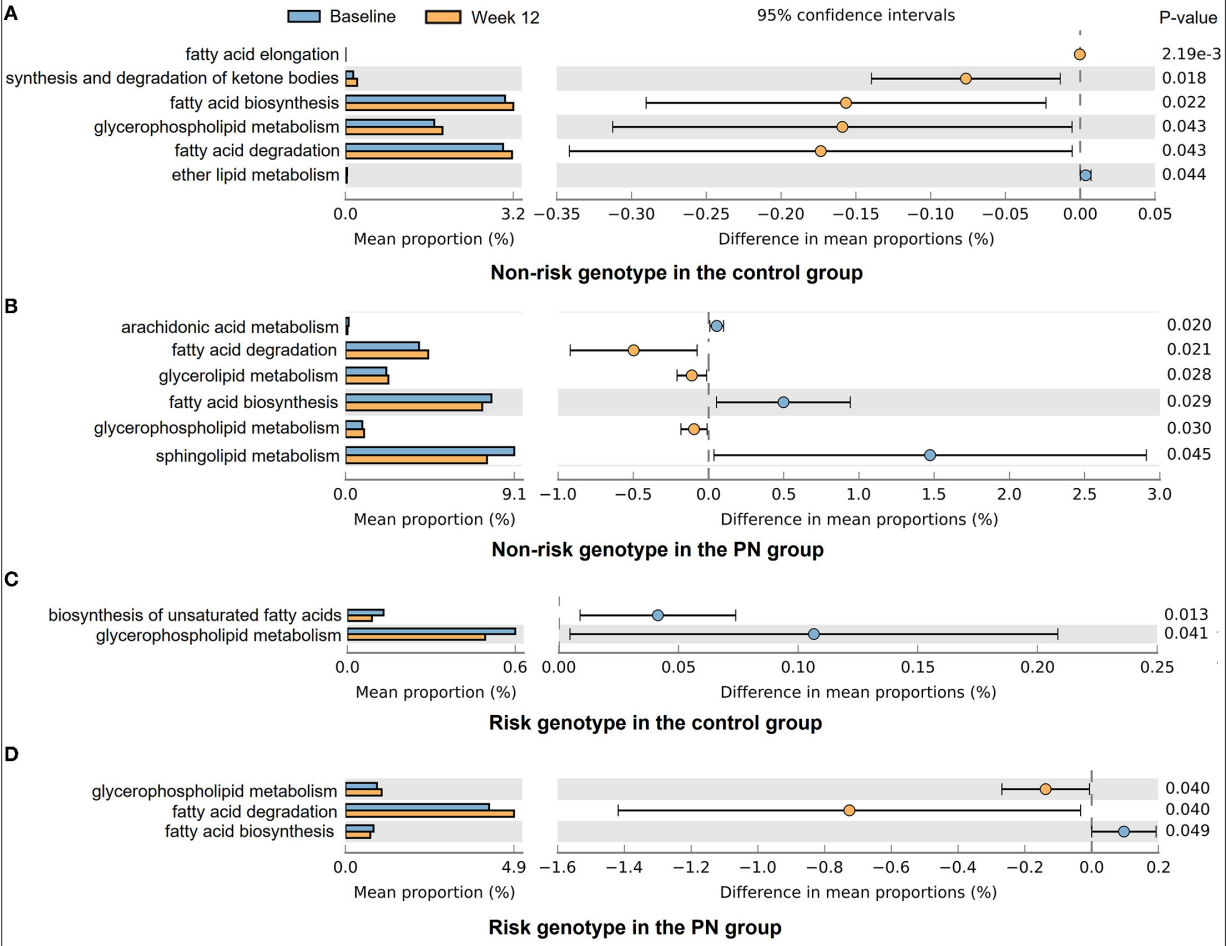
Tax4Fun2 predictions of the altered lipid-related functional composition of gut microbiota under different intervention and genotype groups. The KEGG pathways were analyzed by Tax4Fun2 and shown by STAMP. **(A)** Non-risk genotype in the control group; **(B)** Non-risk genotype in the PN group; **(C)** Risk genotype in the control group; **(D)** Risk genotype in the PN group.

The relative abundance of predicted function (KEGG) between subjects with the non-risk genotype and with the risk genotype of *FTO* at baseline is shown in Supplementary Figure 4. Four pathways related to lipid metabolism including glycerolipid metabolism, glycerophospholipid metabolism, synthesis and degradation of ketone bodies, and fatty acid degradation were identified. Figure 3 showed that except for subjects with the risk genotype in the control group, fatty acid degradation and glycerophospholipid metabolism were significantly increased in the other three subgroups. The fatty acid biosynthesis was significantly decreased under PN intervention regardless of genotype.

## Discussion

To our knowledge, this study firstly explored the association between gut microbiota and different responses to PN intervention in subjects with different genotypes of *FTO*. It was found that the improvement in anthropometric parameters and lipid metabolism was greater in the PN group than in the control group. Subjects with the risk genotype of *FTO* showed greater reduction in anthropometric parameters, TCHO, and LDL than the non-risk subjects. PN intervention effectively increased α diversity of gut microbiota regardless of genotypes. The gut microbiota, which was associated with pathways including fatty acid biosynthesis and degradation, might be involved in lipid metabolism in response to intervention.

The *FTO* gene has been reported to have key roles in regulating energy balance and lipid metabolism process pathways (21). We found that subjects with the risk genotype of *FTO* had lower HDL concentrations and higher LDL concentrations at baseline. Consistently, carriers of homozygotes for the risk allele of *FTO* were found to have 1.25-fold lower HDL cholesterol concentration than carriers of the non-risk genotype in patients with acromegaly (22). In a study of 380 adult Iranian women, the lower levels of HDL were observed in the risk genotypes compared to the non-risk genotype of FTO (23). The mechanism of *FTO* polymorphism affecting lipid metabolism has not been fully understood, but it has been reported that *FTO* could interact with calmodulin-dependent protein kinase II (CaMKII), thereby prolonging CREB phosphorylation and affecting the expression levels of Brain-derived neurotrophic factor (BDNF) and NPY1R neuropeptide Y receptor Y1 (NPY1R), which were related to lipid metabolic process (24). Besides, *FTO* catalyzes the demethylation of m6A to alter the processing, maturation, and translation of the mRNAs of lipid-related genes (25).

Genotypic variation influencing response to nutrition intervention is of growing interest, and some PN intervention approaches have been developed based on genotypes of various genes. We found that subjects with risk genotype of *FTO* had greater anthropometric parameters, TCHO, and LDL amelioration after intervention in our study, indicating the necessity for timely intervention in high-risk populations. Similarly, in a study of 776 high cardiovascular risk subjects aged 55–80 years, subjects with the risk genotype of *FTO* had higher body weight at baseline but lower body weight gain after 3 years of nutritional intervention with a Mediterranean-style-diet (26). After 12 weeks of hydroalcoholic extract of arti-choke supplement in women with metabolic syndrome, those with risk allele of *FTO* showed better improvement in serum TG levels than those without risk allele (27).

In addition, we found significant interaction effects between *FTO* and intervention on anthropometric parameters, TCHO, and LDL after stratifying for specific genotypes of *FTO*, indicating subjects with different genotypes in PN group showed better response to intervention than in the control group. Few studies have explored the interaction between *FTO* and different intervention methods, but the interaction between *FTO* and lifestyle factors has been widely reported. A 6-month RCT of 1,270 adults across seven European countries (Food4Me) study found that moderate-equivalent PA attenuated the effect of *FTO* on BMI (P_interaction_ = 0.020) and WC (P_interaction_ = 0.005) (28). This might be caused by the fact that *FTO* expression in the hypothalamus is regulated by feeding, fasting, and energy restriction, and the *FTO* expression affects energy homeostasis and lipid metabolism (29). In addition, individuals with different FTO genotypes may exhibit different responses to exercise and dietary intervention by altering C-reactive protein (CRP) (30).

The gut microbiota might be partly responsible for the differences in lipid levels in subjects with different genotypes at baseline. *Barnesiellaceae* was the main different gut microbiota between subjects with different genotype of *FTO* at baseline in our study, which plays a crucial role in maintaining health status, including prevent blood stream infection (BSI) and other diseases (31). *Barnesiellaceae* can also serve as a biomarker of obesity and was reported to be higher in control individuals compared to obese patients (32). Among the more abundant gut microbiota in subjects with the *FTO* risk genotype in our study, the *NK4A214_group* had the highest LDA value. A rat experiment showed that the fecal levels of TCHO and TG were positively correlated with the relative abundance of *NK4A214* (33), which might partly account for the higher levels of lipids in subjects with the *FTO* risk genotype at baseline.

Our study showed that α diversity of gut microbiota was significantly increased by PN intervention, which might partly explain the better intervention results in the PN group. The subjects with the non-risk genotype of *FTO* in the control group had minimal change of α diversity after intervention, suggesting that they were less responsive to the intervention. Moreover, the gut microbiota of subjects with different genotypes showed varied responses to the interventions. We found subjects with the risk genotype had greater abundance of *Blautia* after intervention in both PN group and control group, which might partially explain the significant HDL and LDL amelioration in subjects with the risk genotype of *FTO*. *Blautia* is a group of bacteria containing various acetate and butyrate producers (34). *Blautia* was found to be correlated with the improvements in glucose and lipid homeostasis in a RCT study of Type 2 diabetes patients with hyperlipidemia, making it a common target in the management of this disease (34). For subjects with the non-risk genotype, the most significant change in gut microbiota was *Firmicutes*, which included numerous bacterial species involved in butyrate and propionate production, and fatty acid biosynthesis and degradation (35, 36), as proved by KEGG pathway analysis.

To date, studies exploring the association between *FTO* genotype and gut microbiota were still very limited. An animal study showed that *FTO* deficiency mice harbored specific bacterial signature of suppressing inflammation, characterized by higher abundance of *Lactobacillus*, lower *Porphyromonadaceae* and *Helicobacter* (14). It was suggested that *FTO* might target molecules involved in shaping the intestinal microenvironment by regulating the pH value, bile acid metabolism, and immune response (14). Therefore, the gut microbiota might play a modulating role in the different responses to intervention in different *FTO* genotypes.

This RCT study has several strengths. The study subjects were compliant, and the intervention approach was developed by the validated decision trees. *FTO* genotype and gut microbiota were integrated, and multiple analysis methods were used to fully explore factors associated with the individual response to lifestyle interventions. However, this study is still subject to two main limitations. First, the sample size of subjects with TT genotype was relatively small, which might reduce statistical power and increase uncertainty. Second, since gut microbiota was influenced by environmental, dietary, genetic, and other factors, it's difficult to maintain complete homogeneity in different groups of subjects. It should be cautious to interpret our results and apply them to other populations with different lifestyles and diets.

## Conclusions

In conclusion, subjects with the risk genotype of *FTO* showed better response to nutrition intervention in terms of anthropometric and blood lipid parameters. PN intervention resulted in better amelioration of anthropometric parameters, TCHO, and LDL in both genotypes population. The gut microbiota, which were involved in multiple pathways including fatty acid biosynthesis and degradation, might be responsible for the different lipid levels at baseline and modulate different lipid metabolism responses to intervention in different genotypes.

## Data availability statement

The data presented in the study are deposited in the Dryad repository (https://datadryad.org/stash), accession number: https://doi.org/10.5061/dryad.5tb2rbp6t.

## Ethics statement

This trial was approved by the Institutional Review Board (IRB) of the Shanghai Nutrition Society and registered at chictr.org.cn (ChiCTR1900026226). The patients/participants provided their written informed consent to participate in this study. Written informed consent was obtained from the individual(s) for the publication of any potentially identifiable images or data included in this article.

## Author contributions

JZ and JK designed the research. HG conducted the research. JZ, FW, HG, and JD analyzed the data. JZ wrote the manuscript. JK and JC revised the manuscript. JK had primary responsibility for final content. All authors read and approved the final manuscript.

## Conflict of interest

The authors declare that the research was conducted in the absence of any commercial or financial relationships that could be construed as a potential conflict of interest.

## Publisher's note

All claims expressed in this article are solely those of the authors and do not necessarily represent those of their affiliated organizations, or those of the publisher, the editors and the reviewers. Any product that may be evaluated in this article, or claim that may be made by its manufacturer, is not guaranteed or endorsed by the publisher.

## References

[1] Roth GA Mensah GA Johnson CO Addolorato G Ammirati E Baddour LM. Global burden of cardiovascular diseases and risk factors, 1990-2019: update from the Gbd 2019 study. J Am Coll Cardiol. (2020) 76:2982–3021. 10.1016/j.jacc.2020.11.010 33309175 PMC7755038

[2] Zhou M Wang H Zeng X Yin P Zhu J Chen W. Mortality, morbidity, and risk factors in China and its provinces, 1990-2017: a systematic analysis for the global burden of disease study 2017. Lancet. (2019) 394:1145–58. 10.1016/S0140-6736(19)30427-1 31248666 PMC6891889

[3] Poveda A Chen Y Brandstrom A Engberg E Hallmans G Johansson I. The heritable basis of gene-environment interactions in cardiometabolic traits. Diabetologia. (2017) 60:442–52. 10.1007/s00125-016-4184-0 28004149 PMC6518092

[4] Yu E Malik VS Hu FB. Cardiovascular disease prevention by diet modification: jacc health promotion series. J Am Coll Cardiol. (2018) 72:914–26. 10.1016/j.jacc.2018.02.085 30115231 PMC6100800

[5] Stover PJ King JC. More nutrition precision, better decisions for the health of our nation. J Nutr. (2020) 150:3058–60. 10.1093/jn/nxaa280 33025003 PMC7919333

[6] Gardner CD Trepanowski JF Del Gobbo LC Hauser ME Rigdon J Ioannidis JPA. Effect of low-fat vs low-carbohydrate diet on 12-month weight loss in overweight adults and the association with genotype pattern or insulin secretion: the dietfits randomized clinical trial. JAMA. (2018) 319:667–79. 10.1001/jama.2018.0245 29466592 PMC5839290

[7] Stanton MV Robinson JL Kirkpatrick SM Farzinkhou S Avery EC Rigdon J. Dietfits study (diet intervention examining the factors interacting with treatment success) - study design and methods. Contemp Clin Trials. (2017) 53:151–61. 10.1016/j.cct.2016.12.021 28027950 PMC5274550

[8] Oyeyemi BF Ologunde CA Olaoye AB Alamukii NA. Fto gene associates and interacts with obesity risk, physical activity, energy intake, and time spent sitting: pilot study in a nigerian population. J Obes. (2017) 2017:3245270. 10.1155/2017/3245270 28607773 PMC5457775

[9] Jonsson A Renstrom F Lyssenko V Brito EC Isomaa B Berglund G. Assessing the effect of interaction between an fto variant (Rs9939609) and physical activity on obesity in 15,925 Swedish and 2,511 Finnish adults. Diabetologia. (2009) 52:1334–8. 10.1007/s00125-009-1355-2 19373445

[10] Dwivedi AK Dubey P Cistola DP Reddy SY. Association between obesity and cardiovascular outcomes: updated evidence from meta-analysis studies. Curr Cardiol Rep. (2020) 22:25. 10.1007/s11886-020-1273-y 32166448 PMC12285736

[11] Xiang L Wu H Pan A Patel B Xiang G Qi L. Fto Genotype and weight loss in diet and lifestyle interventions: a systematic review and meta-analysis. Am J Clin Nutr. (2016) 103:1162–70. 10.3945/ajcn.115.123448 26888713 PMC4807705

[12] Hughes RL Marco ML Hughes JP Keim NL Kable ME. The role of the gut microbiome in predicting response to diet and the development of precision nutrition models-Part I: overview of current methods. Adv Nutr. (2019) 10:953–78. 10.1093/advances/nmz022 31225589 PMC6855943

[13] Kovatcheva-Datchary P Nilsson A Akrami R Lee YS De Vadder F Arora T. Dietary fiber-induced improvement in glucose metabolism is associated with increased abundance of prevotella. Cell Metab. (2015) 22:971–82. 10.1016/j.cmet.2015.10.001 26552345

[14] Sun L Ma L Zhang H Cao Y Wang C Hou N. Fto deficiency reduces anxiety- and depression-like behaviors in mice via alterations in gut microbiota. Theranostics. (2019) 9:721–33. 10.7150/thno.31562 30809304 PMC6376469

[15] Kan J Ni J Xue K Wang F Zheng J Cheng J. Personalized nutrition intervention improves health status in overweight/obese chinese adults: a randomized controlled trial. Front Nutr. (2022) 9:919882. 10.3389/fnut.2022.919882 35811975 PMC9258630

[16] Patterson E. Guidelines for Data Processing and Analysis of the International Physical Activity Questionnaire (Ipaq)-Short and Long Forms (2005).

[17] Celis-Morales C Livingstone KM Marsaux CF Macready AL Fallaize R O'Donovan CB. Effect of personalized nutrition on health-related behaviour change: evidence from the food4me european randomized controlled trial. Int J Epidemiol. (2017) 46:578–88. 10.1093/ije/dyw186 27524815

[18] Chiang KM Chang HC Yang HC Chen CH Chen HH Lee WJ. Genome-wide association study of morbid obesity in han Chinese. BMC Genet. (2019) 20:97. 10.1186/s12863-019-0797-x 31852448 PMC6921553

[19] Chen X Gao Y Yang X Zhang H Mo Z Tan A. Relationship of Fto gene variations with nafld risk in Chinese Men. Open Life Sci. (2020) 15:860–7. 10.1515/biol-2020-0081 33817272 PMC7874577

[20] Cao M Zhang L Chen T Shi A Xie K Li Z. Genetic susceptibility to gestational diabetes mellitus in a Chinese population. Front Endocrinol. (2020) 11:247. 10.3389/fendo.2020.00247 32390949 PMC7188786

[21] Lan N Lu Y Zhang Y Pu S Xi H Nie X. Fto - a common genetic basis for obesity and cancer. Front Genet. (2020) 11:559138. 10.3389/fgene.2020.559138 33304380 PMC7701174

[22] Franczak A Kolackov K Jawiarczyk-Przybylowska A Bolanowski M. Association between Fto gene polymorphisms and Hdl cholesterol concentration may cause higher risk of cardiovascular disease in patients with acromegaly. Pituitary. (2018) 21:10–5. 10.1007/s11102-017-0840-8 28913579 PMC5767210

[23] Jalili V Mokhtari Z Rastgoo S Hajipour A Bourbour F Gholamalizadeh M. The association between Fto Rs9939609 polymorphism and serum lipid profile in adult women. Diabetol Metab Syndr. (2021) 13:138. 10.1186/s13098-021-00754-0 34801066 PMC8606052

[24] Marcinkowski M Pilzys T Garbicz D Piwowarski J Przygonska K Winiewska-Szajewska M. Calmodulin as Ca(2+)-dependent interactor of Fto dioxygenase. Int J Mol Sci. (2021) 22:10869. 10.3390/ijms221910869 34639211 PMC8509707

[25] Yang Z Yu GL Zhu X Peng TH Lv YC. Critical roles of Fto-mediated Mrna M6a demethylation in regulating adipogenesis and lipid metabolism: implications in lipid metabolic disorders. Genes Dis. (2022) 9:51–61. 10.1016/j.gendis.2021.01.005 35005107 PMC8720706

[26] Razquin C Martinez JA Martinez-Gonzalez MA Bes-Rastrollo M Fernandez-Crehuet J Marti A. 3-year intervention with a mediterranean diet modified the association between the Rs9939609 gene variant in Fto and body weight changes. Int J Obes. (2010) 34:266–72. 10.1038/ijo.2009.233 19918250

[27] Rezazadeh K Rahmati-Yamchi M Mohammadnejad L Ebrahimi-Mameghani M Delazar A. Effects of artichoke leaf extract supplementation on metabolic parameters in women with metabolic syndrome: influence of Tcf7l2-Rs7903146 and Fto-Rs9939609 polymorphisms. Phytother Res. (2018) 32:84–93. 10.1002/ptr.5951 29193419

[28] Celis-Morales C Marsaux CF Livingstone KM Navas-Carretero S San-Cristobal R O'Donovan C B. Physical activity attenuates the effect of the Fto genotype on obesity traits in european adults: the food4me study. Obesity. (2016) 24:962–9. 10.1002/oby.21422 26921105

[29] Liu SJ Tang HL He Q Lu P Fu T Xu XL. Fto is a transcriptional repressor to auto-regulate its own gene and potentially associated with homeostasis of body weight. J Mol Cell Biol. (2019) 11:118–32. 10.1093/jmcb/mjy028 29771336 PMC6734146

[30] Zou ZC Mao LJ Shi YY Chen JH Wang LS Cai W. Effect of exercise combined with dietary intervention on obese children and adolescents associated with the Fto Rs9939609 polymorphism. Eur Rev Med Pharmacol Sci. (2015) 19:4569-75. 26698254

[31] Cui J Yang X Wang F Liu S Han S Chen B. Effects of ammonia on growth performance, lipid metabolism and cecal microbial community of rabbits. PLoS ONE. (2021) 16:e0252065. 10.1371/journal.pone.0252065 34191811 PMC8244895

[32] Del Chierico F Abbatini F Russo A Quagliariello A Reddel S Capoccia D. Gut microbiota markers in obese adolescent and adult patients: age-dependent differential patterns. Front Microbiol. (2018) 9:1210. 10.3389/fmicb.2018.01210 29922272 PMC5996250

[33] Zhang Q Fan XY Cao YJ Zheng TT Cheng WJ Chen LJ. The beneficial effects of lactobacillus brevis Fzu0713-fermented laminaria japonica on lipid metabolism and intestinal microbiota in hyperlipidemic rats fed with a high-fat diet. Food Funct. (2021) 12:7145–60. 10.1039/D1FO00218J 34231612

[34] Tong X Xu J Lian F Yu X Zhao Y Xu L. Structural alteration of gut microbiota during the amelioration of human type 2 diabetes with hyperlipidemia by metformin and a traditional chinese herbal formula: a multicenter, randomized, open label clinical trial. mBio. (2018) 9:e02392–1. 10.1128/mBio.02392-17 29789365 PMC5964358

[35] Louis P Flint HJ. Formation of propionate and butyrate by the human colonic microbiota. Environ Microbiol. (2017) 19:29–41. 10.1111/1462-2920.13589 27928878

[36] Feng W Ao H Peng C. Gut microbiota, short-chain fatty acids, and herbal medicines. Front Pharmacol. (2018) 9:1354. 10.3389/fphar.2018.01354 30532706 PMC6265305

