# Supplementary material for: Gut microbiota modulates differential lipid metabolism outcomes associated with FTO gene polymorphisms in response to personalized nutrition intervention
Source: Front Nutr. 2022 Sep 15;9:985723. doi: 10.3389/fnut.2022.985723 (PMC9520577; doi:10.3389/fnut.2022.985723)
Supplement: Supplementary file 1 [file Table_1.DOCX]

Supplementary Material

**Table S1 Dietary intake and physical activity at baseline and week 12 in different genotype groups**

| **Factor** | **Time** | **PN group** | | |  | **Control group** | | |
| --- | --- | --- | --- | --- | --- | --- | --- | --- |
|  |  | Non-risk genotype  (N=112) | Risk genotype  (N=54) | p-value |  | Non-risk genotype  (N=98) | Risk genotype  (N=54) | p-value |
| **Total energy intake, kcal/day** | Baseline | 1429.0 ^a^ | 1663.0 | 0.149 |  | 1503.0 | 1444.5 | 0.846 |
|  | Week 12 | 1392.5 | 1438.0 | 0.328 |  | 1478.5 | 1514.5 | 0.705 |
|  | P-value | 0.243 | 0.428 |  |  | 0.369 | 0.776 |  |
| **Protein intake, g/day** | Baseline | 61.1 | 71.3 | 0.168 |  | 64.1 | 62.2 | 0.411 |
|  | Week 12 | 61.8 | 69.9 | 0.378 |  | 65.7 | 59.6 | 0.673 |
|  | P-value | 0.947 | 0.506 |  |  | 0.532 | 0.355 |  |
| **Fat intake, g/day** | Baseline | 60.2 | 57.1 | 0.821 |  | 62.6 | 60.2 | 0.643 |
|  | Week 12 | 48.0 | 52.9 | 0.454 |  | 55.7 | 58.0 | 0.810 |
|  | P-value | **0.007** | 0.312 |  |  | **0.029** | 0.537 |  |
| **Carbohydrate intake, g/day** | Baseline | 155.4 | 170.9 | 0.205 |  | 155.9 | 172.7 | 0.309 |
|  | Week 12 | 152.8 | 161.4 | 0.565 |  | 161.3 | 165.7 | 0.853 |
|  | P-value | 0.848 | 0.916 |  |  | 0.545 | 0.470 |  |
| **Total MET, min/week** | Baseline | 2326.0 | 1815.0 | 0.211 |  | 2721.0 | 1551.0 | **0.008** |
|  | Week 12 | 3119.0 | 2535.0 | 0.623 |  | 1758.0 | 1746.0 | 0.962 |
|  | P-value | **0.008** | **0.024** |  |  | **0.001** | 0.779 |  |
| **Steps per day** | Baseline | 8068.5 | 8241.5 | 0.934 |  | 8679.5 | 8214.5 | 0.250 |
|  | Week 12 | 9648.2 | 10086.4 | 0.828 |  | 8468.5 | 8488.7 | 0.969 |
|  | P-value | **<0.001** | **0.001** |  |  | 0.694 | 0.168 |  |

a: median; Group difference was evaluated using ANOVA after Ln transformation; the difference at week 12 was evaluated using ANCOVA with adjustment for the baseline measures; within group change from baseline to week 12 was evaluated using paired t-test.

**Table S2** **Associations between genotype and intervention with change of anthropometric parameters and lipids from baseline to week 12 by GLM**

| **Factor** | **Model** | **Genotype** | **Intervention** | **Interaction** |
| --- | --- | --- | --- | --- |
| **Anthropometric parameters** | | |  |  |
| **Δ_Weight_** | Model 1 | -0.917 (-1.39~-0.421)** | -0.940 (-1.48~-0.382)** |  |
|  | Model 2 | -0.884 (-1.40~-0.410)** | -0.937 (-1.50~-0.388)** |  |
|  | Model 3 | -0.884 (-1.38~-0.397)** | -0.947 (-1.49~-0.438)** | -0.032 (-0.550~0.474) |
| **Δ_BMI_** | Model 1 | -0.329 (-0.509~-0.143)** | -0.334 (-0.538~-0.123)** |  |
|  | Model 2 | -0.324 (-0.509~-0.154)** | -0.341 (-0.547~-0.132)** |  |
|  | Model 3 | -0.325 (-0.509~-0.153)** | -0.338 (-0.538~-0.152)** | 0.009 (-0.189~0.197) |
| **Δ_WC_** | Model 1 | -0.943 (-1.44~-0.452)** | -1.21 (-1.71~-0.709)** |  |
|  | Model 2 | -0.866 (-1.38~-0.368)** | -1.21 (-1.71~-0.685)** |  |
|  | Model 3 | -0.871 (-1.38~-0.384)** | -1.10 (-1.62~-0.584)** | 0.343 (-0.146~0.814) |
| **Δ_fat percent_** | Model 1 | -0.430 (-0.732~-0.128)* | -0.764 (-1.08~-0.435)** |  |
|  | Model 2 | -0.416 (-0.726~-0.108)* | -0.757 (-1.06~-0.458)** |  |
|  | Model 3 | -0.418 (-0.718~-0.110)* | -0.734 (-1.05~-0.446)** | 0.072 (-0.216~0.352) |
| **Blood lipids** | | | | |
| **Δ_Total cholesterol_** | Model 1 | -0.168 (-0.284~-0.062)** | -0.170 (-0.280~-0.067)** |  |
|  | Model 2 | -0.180 (-0.295~-0.077)** | -0.155 (-0.258~-0.053)** |  |
|  | Model 3 | -0.181 (-0.294~-0.077)** | -0.138 (-0.246~-0.034)** | 0.054 (-0.049~0.157) |
| **Δ_TG_** | Model 1 | -0.036 (-0.147~0.070) | -0.147 (-0.256~-0.029)** |  |
|  | Model 2 | -0.019 (-0.119~0.079) | -0.084 (-0.170~0.000)* |  |
|  | Model 3 | -0.018 (-0.117~0.082) | -0.089 (-0.181~0.000)* | -0.015 (-0.104~0.067) |
| **Δ_HDL_** | Model 1 | 0.033 (-0.012~0.076) | 0.040 (-0.008~0.082) |  |
|  | Model 2 | 0.023 (-0.021~0.065) | 0.040 (-0.005~0.082) |  |
|  | Model 3 | 0.023 (-0.021~0.065) | 0.036 (-0.011~0.080) | -0.014 (-0.059~0.031) |
| **Δ_LDL_** | Model 1 | -0.128 (-0.206~-0.044)** | -0.106 (-0.190~-0.013)* |  |
|  | Model 2 | -0.122 (-0.200~-0.044)** | -0.104 (-0.184~-0.018)* |  |
|  | Model 3 | -0.123 (-0.200~-0.043)** | -0.095 (-0.171~-0.014)* | 0.028 (-0.054~0.104) |

Data were presented as β and 95% CI. The non-risk genotype and control group were set as reference. Model 1 only included genotype and intervention group as independent variables, Model 2 further adjusted for age, BMI, intake of macronutrients, total MET, and anthropometric or lipid measurements at baseline, and Model 3 further included interaction terms of genotype and intervention. 95% CI of coefficients was generated by 1000 times of bootstrap. *: p<0.05; **: p<0.01.

**Figure S1. Comparison of gut microbiota composition between subjects with the risk and non-risk genotype of FTO at baseline.** A: Taxonomic representation of statistically and biologically consistent differences between non-risk (red) and risk (green) shown in cladogram by LefSe analysis. Differences are represented by the color of the most abundant class. Dot size is proportional to the abundance of the taxon. B: Histogram of the LDA scores that are differentially abundant between risk and non-risk genotype of FTO. Length indicates the effect size associated with a taxon, and only taxa with an LDA significant threshold >2 and p<0.05 are shown. LDA: Linear discriminant analysis.

**Figure S2 Comparison of gut microbiota composition in subjects with the non-risk genotype of FTO before and after PN or control intervention.** Histogram of the LDA scores that are differentially abundant between baseline and Week 12 after control (A) or PN (B) intervention. Length indicates the effect size associated with a taxon, and only taxa with an LDA significant threshold >2 and p<0.05 are shown. LDA: Linear discriminant analysis.

**Figure S3 Comparison of gut microbiota composition in subjects with the risk genotype of FTO before and after PN or control intervention.** Histogram of the LDA scores that are differentially abundant between between baseline and Week 12 after control (A) or PN (B) intervention. Length indicates the effect size associated with a taxon, and only taxa with an LDA significant threshold >2 and p<0.05 are shown. LDA: Linear discriminant analysis.

**Figure S4** **Tax4Fun2 predictions of the significantly differential lipid-related functional composition of gut microbiota at baseline between risk and non-risk genotype of FTO.** The KEGG pathways were analyzed by Tax4Fun2 and shown by STAMP.

**Figure S1**


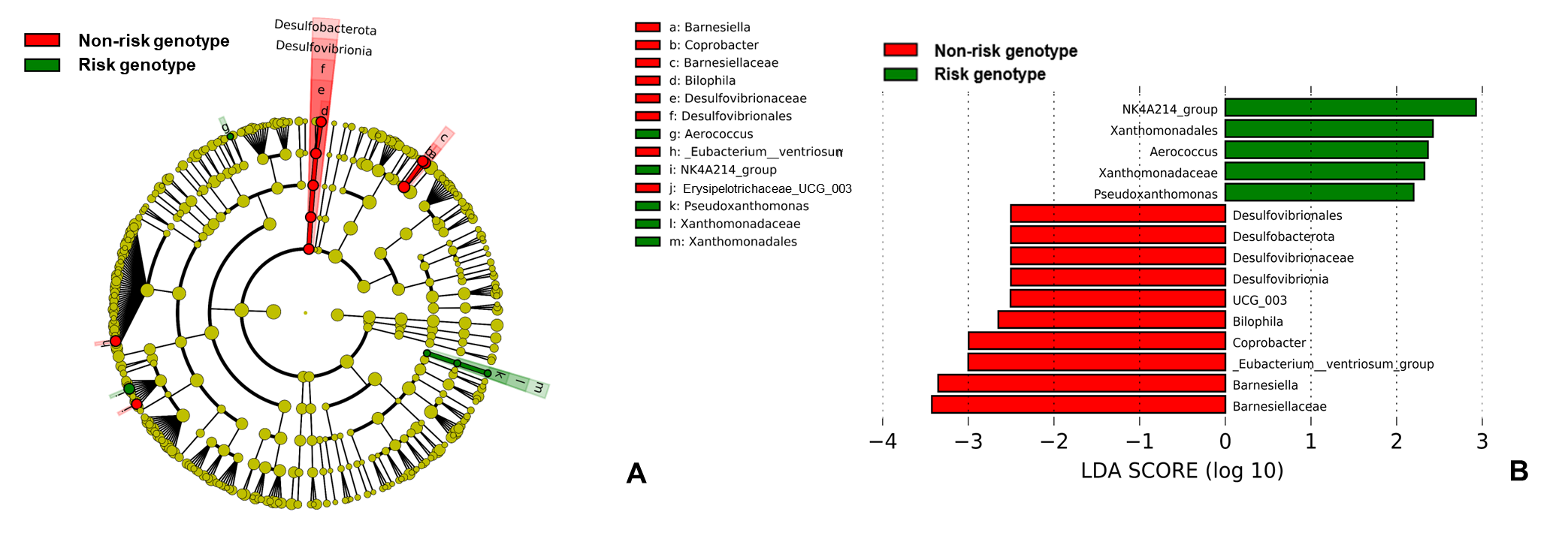


**Figure S2**

**
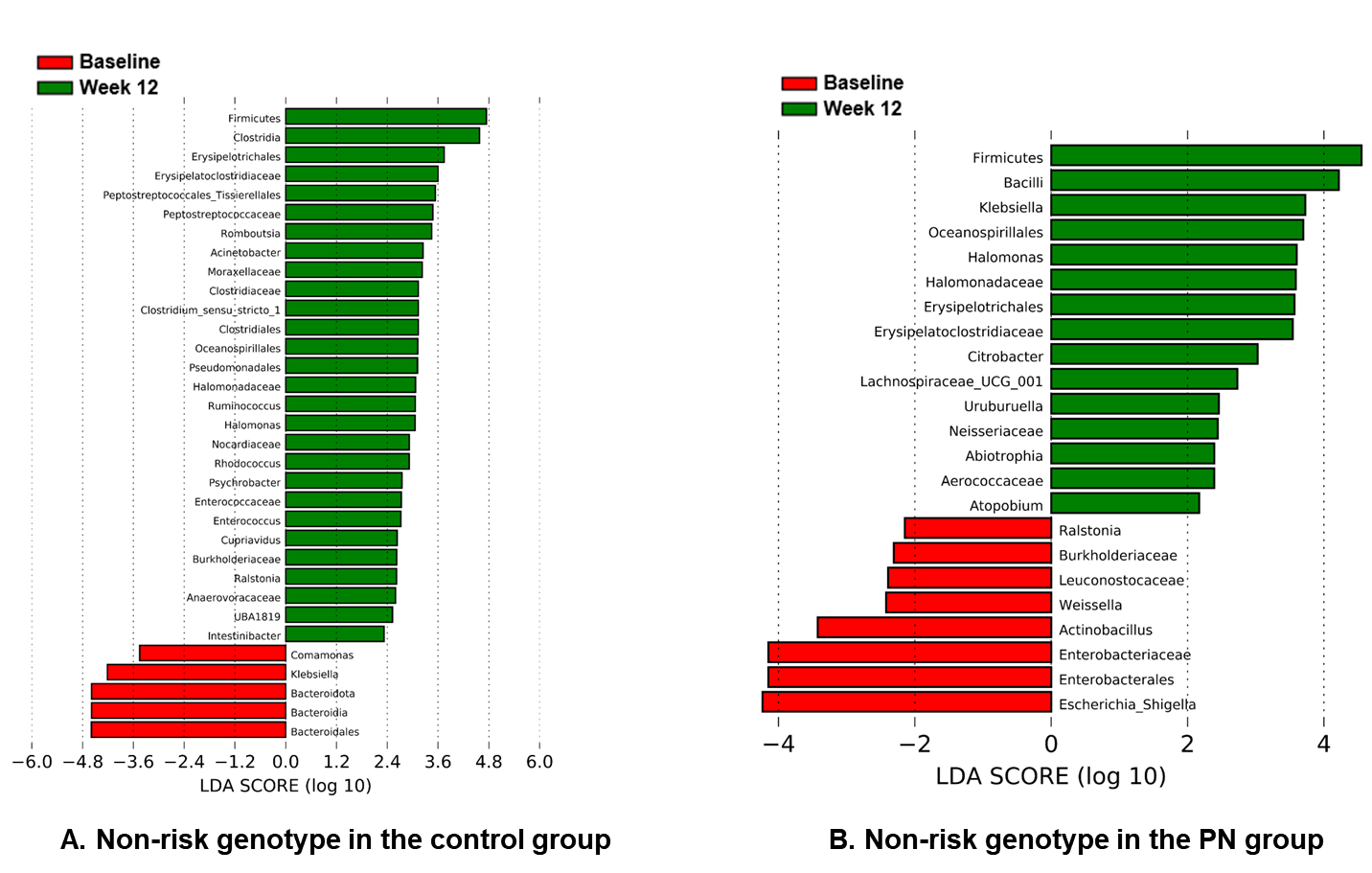
**

**Figure S3**

**
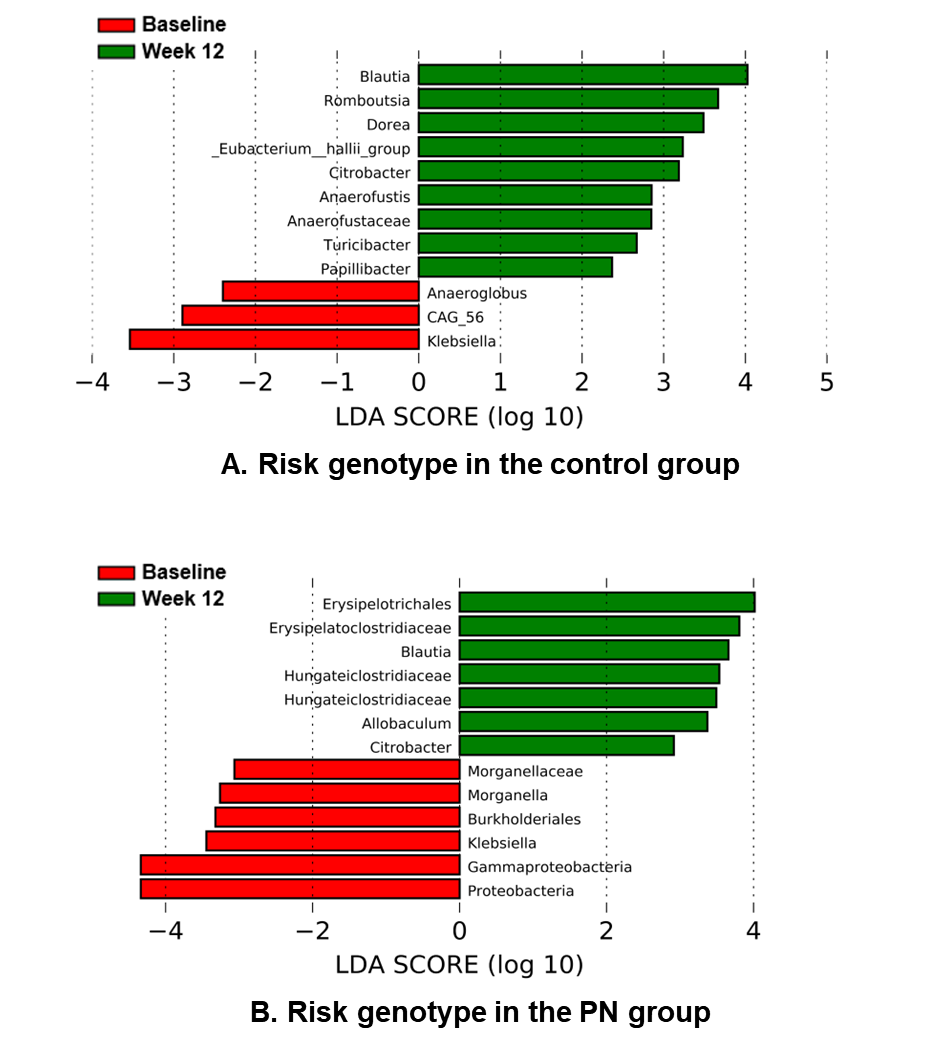
**

**Figure S4**

**
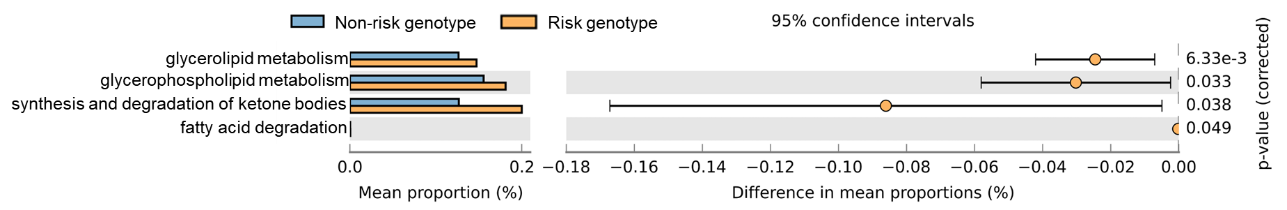
**
